# Case Report: Mavacamten in mid-ventricular and apical hypertrophic cardiomyopathy—a case of targeted therapy beyond left ventricular outflow tract obstruction

**DOI:** 10.3389/fmed.2026.1807582

**Published:** 2026-03-30

**Authors:** Lijia Yang, Hongyan Dai, Yuanyuan Rong

**Affiliations:** Department of Cardiology, Qingdao Hospital, University of Health and Rehabilitation Sciences (Qingdao Municipal Hospital), Qingdao, Shandong, China

**Keywords:** case report, echocardiography, hypertrophic cardiomyopathy, mavacamten, mid-ventricular obstruction

## Abstract

**Background:**

Mavacamten targets cardiac myosin ATPase, reducing the formation of actin-myosin cross-bridges, which alleviates excessive myocardial contraction and improves diastolic function. It has been approved for patients with hypertrophic cardiomyopathy symptomatic for left ventricular outflow tract obstruction. Nevertheless, data on the use of mavacamten in patients with mid-ventricular obstruction and apical hypertrophic cardiomyopathy are limited.

**Case summary:**

This patient is a 56-year-old female presenting with chest pain and unexplained syncope, along with mid-ventricular obstruction and apical hypertrophic cardiomyopathy. Echocardiography showed a mid-ventricular peak gradient of 115 mmHg. Her symptoms were relieved after treatment with mavacamten, and at the 32-week follow-up after treatment, the mid-left ventricular cavity peak pressure gradient was reduced to 34 mmHg.

**Significance:**

In this case, mavacamten improves mid-ventricular obstruction and alleviates symptoms simultaneously. The symptomatic improvement achieved by mavacamten treatment is not limited to hypertrophic cardiomyopathy complicated with left ventricular outflow tract obstruction; it is also effective for a specific type of hypertrophic cardiomyopathy, namely mid-left ventricular obstruction.

## Introduction

Hypertrophic cardiomyopathy (HCM) is a myocardial disease characterized by asymmetric myocardial hypertrophy, with a global prevalence of approximately 1 in 500 (1). It is one of the most common inherited cardiovascular diseases. The most common pathogenic cause is autosomal dominant mutations in the *MYH7* gene, which encodes the sarcomeric protein *β*-myosin heavy chain, and the *MYBPC3* gene (myosin-binding protein C) (2). Based on hemodynamic characteristics, HCM is classified into obstructive hypertrophic cardiomyopathy and non-obstructive hypertrophic cardiomyopathy (3).

Mid-ventricular obstruction (MVO) is a special phenotype of HCM, and there is a paucity of literature on MVO. Efthimiadis et al. (4) conducted a 7-year study on 423 HCM patients and identified 34 cases with MVO. The MVO group had higher incidences of endpoint events such as all-cause mortality, cardiovascular (CV) mortality, and sudden cardiac death. Moreover, 11.8% of MVO patients progressed to end-stage heart failure, and 26.5% developed left ventricular apical aneurysms.

Mavacamten is a selective and reversible inhibitor of cardiac myosin ATPase. It reversibly inhibits the excessive formation of myosin-actin cross-bridges, thereby suppressing excessive myocardial contraction, improving diastolic compliance and energy metabolism. It has been approved by the U.S. Food and Drug Administration (FDA) and the European Medicines Agency (EMA) for the treatment of adult symptomatic hypertrophic obstructive cardiomyopathy (HOCM) patients with New York Heart Association (NYHA) Class II-III heart failure. Both European and American guidelines for the management of HCM patients recommend the use of myosin inhibitors for HOCM (as Class IIa and Class Ib recommendations, respectively) (3, 5). There are only a few case reports on the use of myosin inhibitors in treating symptomatic MVO patients, and reports on mavacamten in treating MVO patients without left ventricular outflow tract obstruction are extremely rare.

This paper reports a case of a patient with apical hypertrophy, diffuse left ventricular wall hypertrophy complicated by MVO, who was admitted to hospital due to chest pain accompanied by unexplained syncope. Transthoracic echocardiography on admission revealed a peak pressure gradient of 115 mmHg in the mid-ventricle, a marked elevation of B-type natriuretic peptide (BNP) to 778.3 pg./mL (normal value: < 100 pg./mL), and a high-sensitivity cardiac troponin I (hs-cTnI) level of 0.062 ng/mL (normal value: < 0.016 ng/mL). After careful evaluation, the patient was treated with mavacamten, and follow-up outpatient examinations showed resolution of MVO.

## Case presentation

A 56-year-old female patient, who was diagnosed with HCM 8 months ago, has no history of coronary heart disease, hypertension or diabetes, nor a family history of sudden death. She was admitted to the hospital due to intermittent chest pain for 1 year and a sudden syncope episode.

Upon admission, the patient’s routine examinations showed normal body temperature, blood pressure of 125/67 mmHg, heart rate of 67 beats per minute, and respiratory rate of 18 breaths per minute. Cardiac auscultation revealed a regular rhythm, and a grade 2 systolic murmur was audible at the 3rd and 4th intercostal spaces along the left sternal border. Pulmonary auscultation indicated clear breath sounds without any moist or dry rales. Mild pitting edema was noted in both lower extremities.

The patient’s electrocardiogram (ECG) showed T-wave inversion in leads I, II, aVL, aVF, and V2-V6. The 24-h ambulatory electrocardiogram (Holter ECG) revealed no rhythm pauses lasting more than 3 s, and no episodes of ventricular tachycardia or non-sustained ventricular tachycardia were recorded.

A transthoracic echocardiogram (TTE) performed on the day of admission revealed the following findings: diffuse thickening of the left ventricular wall, predominantly involving the mid-left ventricular segment and apex, with the maximum thickness measuring approximately 22.5 mm. During systole, there was near-occlusion of the mid-cavity and apical regions of the left ventricular chamber, leading to an increased blood flow velocity of about 5.4 m/s and a peak pressure gradient of approximately 115 mmHg after Valsalva manoeuvre, along with dynamic obstruction noted in systole. No significant stenosis was observed in the left ventricular outflow tract (LVOT), and no systolic anterior motion (SAM) of the mitral valve was detected. The thickness of the remaining ventricular walls was normal. Three-dimensional echocardiography showed a left ventricular ejection fraction of 58% (LVEF = 58%).

Laboratory tests showed normal results of complete blood count, electrolytes, and liver function tests. BNP was significantly elevated at 778.3 pg./mL (normal value < 100 pg./mL), and hs-cTnI level was 0.062 ng/mL (normal value < 0.016 ng/mL). Laboratory tests performed 8 months prior revealed normal levels of serum free light chains and serum ferritin. To determine whether myocardial hypertrophy was caused by Fabry disease, *α*-galactosidase assay was conducted, with a negative result. Technetium-99 m pyrophosphate (^99^ᵐTc-PYP) scintigraphy results ruled out transthyretin (ATTR)-type cardiac amyloidosis.

The patient presented with intermittent chest pain that was not associated with physical activity. To clarify the status of coronary artery lesions, coronary computed tomography angiography was performed, which revealed no significant stenosis of the coronary arteries. Further cardiopulmonary exercise testing showed no obvious dynamic changes on the electrocardiogram, and the pulmonary function was normal.

Cardiac magnetic resonance (CMR) imaging revealed left ventricular (LV) myocardial hypertrophy, near-occlusion of the mid-cavity and apical regions of the left ventricular chamber during systole, and diffuse late gadolinium enhancement (LGE) in the left ventricle.

Analysis of the cardiovascular genetic testing panel identified a variant in the *MYPN* gene (NM_032578.4, c.662A > G; p.Arg208Gly), which was classified as a variant of uncertain significance in accordance with the guidelines of the American College of Medical Genetics and Genomics.

Eight months ago, the patient received treatment with a beta-blocker, a sodium-glucose cotransporter 2 inhibitor, and a calcium channel blocker, yet no significant improvement in symptoms was observed. After careful consideration, although the patient had no left ventricular outflow tract obstruction, combined with the mechanism of action of mavacamten, the drug might alleviate mid-left ventricular cavity obstruction. Following full communication with the patient, treatment with mavacamten at a dose of 2.5 mg once daily was initiated.

**Figure 1:**
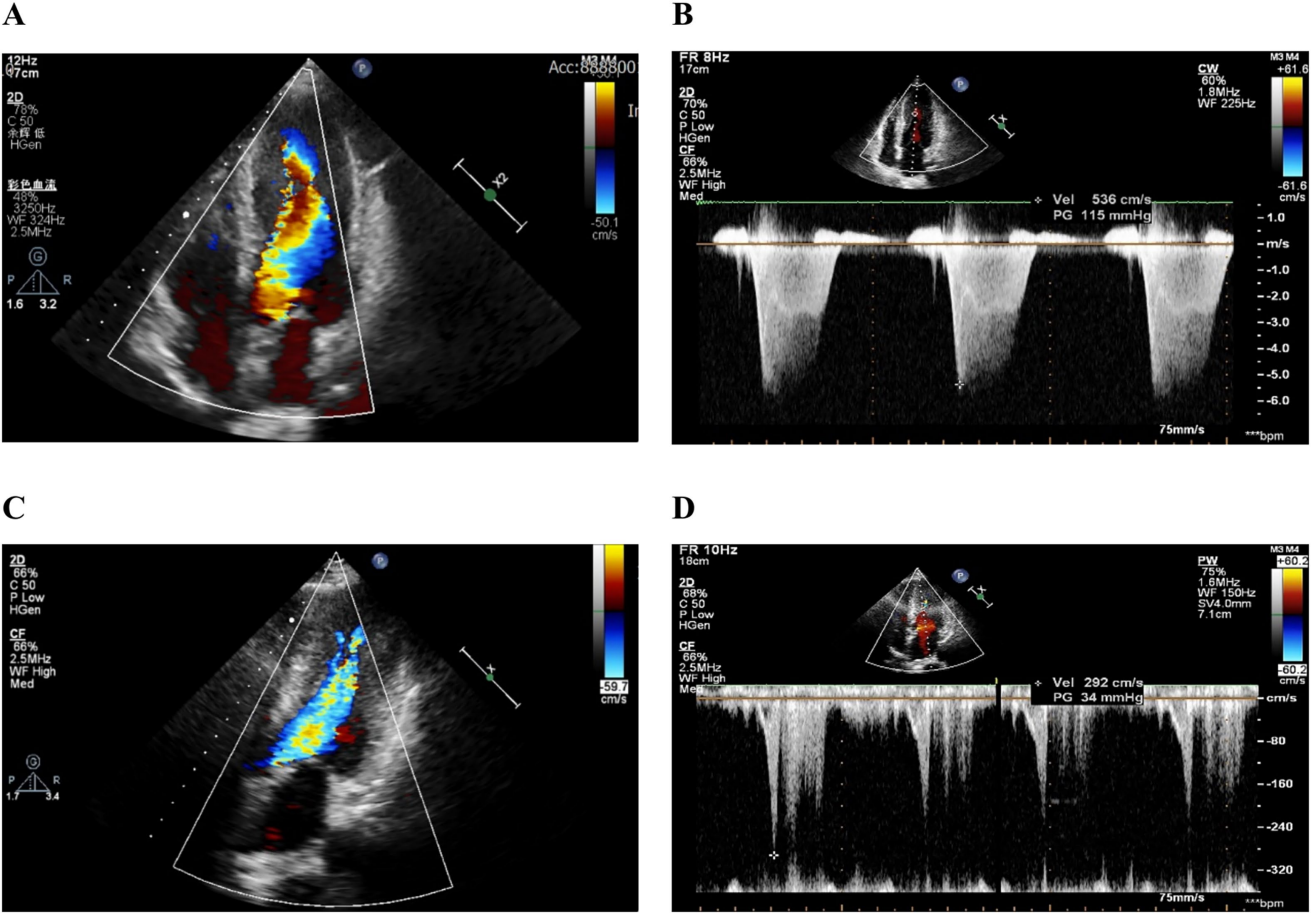
Echocardiographic characteristics at baseline and at 32 weeks follow-up. Color Doppler showing mid-ventricular acceleration before and after mavacamten treatment **(A,C)**; Peak mid-ventricular gradient before and after mavacamten treatment **(B,D)**.

At the 4-week outpatient follow-up, the patient’s symptoms and quality of life had improved significantly, and the BNP level had decreased to 252 pg./mL. A repeat echocardiogram showed no significant reduction in the peak pressure gradient in the mid-left ventricular cavity, and the left ventricular ejection fraction (LVEF) remained unchanged. The patient continued mavacamten treatment. After 12 weeks of treatment, the patient was advised to increase the dosage to 5 mg once daily. By the 20-week mark, laboratory tests indicated a normal BNP level; the patient’s activity tolerance had increased markedly, with no obvious discomfort. A follow-up echocardiogram revealed a peak pressure gradient of 81 mmHg in the mid-left ventricular cavity. Subsequent follow-up recommendations included a further dosage increase, which the patient declined due to the high cost of the medication. Throughout the treatment period, the patient did not experience recurrent syncope, chest pain, palpitations, or other adverse symptoms. At the 32-week follow-up, a repeat echocardiogram demonstrated a peak pressure gradient of 34 mmHg in the mid-left ventricular cavity. The echocardiographic characteristics at baseline and 32 weeks after mavacamten treatment are shown in Figures 1A–D. Detailed comparative measurements are presented in Table 1.

**Table 1:** Clinical and echocardiographic characteristics of patient at baseline, 4, 8, 12, 16, 20 and 32 weeks follow-up.

Variables	Baseline	4 weeks	8 weeks	12 weeks	16 weeks	20 weeks	32 weeks
Mavacamten dose(mg/day)	2.5	2.5	2.5	2.5	5	5	5
NYHA class	II-III	II	II	II	II	II	II
Maximum wall thickness (mm)	22.5	22.7	22.3	22	22.5	22	22
Peak mid-cavity gradient (mmHg)	115	107	76	102	108	81	34
Left ventricular ejection fraction (LVEF %)	58	58	58	60	58	60	58
E/e′	11.1	13.4	9.1	10.5	9.7	13	10.5
Systolic anterior motion (SAM)	—	—	—	—	—	—	—
BNP (pg/mL)	778.3		252			66	
Hs-cTnI (ng/mL)	0.062		0.077			0.068	

## Discussion

This case represents a rare subtype of HCM, specifically apical hypertrophic cardiomyopathy complicated by mid-left ventricular hypertrophy and MVO, which confirms that mavacamten exerts a significant therapeutic effect on MVO in patients with HCM.

HCM is classified by the location of obstruction into the following types: left ventricular outflow tract obstruction, mid-left ventricular obstruction (MVO), subaortic obstruction, and right ventricular outflow tract obstruction. Among these, MVO may be caused by the anomalous insertion of the anterior papillary muscle, excessive hypertrophy of the mid-ventricle, or pathological arrangement of the papillary muscles themselves (6). With the clinical application of CMR, myocardial hypertrophy at different locations has been clearly identified and classified. Apical hypertrophic cardiomyopathy was first described by Sakamoto et al. (7) and is often prone to missed diagnosis without the auxiliary diagnosis of CMR. Some patients with apical hypertrophic cardiomyopathy have concomitant hypertrophy of the mid-left ventricular segment, in which case MVO complicated by cavitary obliteration may occur (6). The presence of an apical ventricular aneurysm indicates a poor prognosis. Such patients carry a higher risk of complications undergoing myectomy, and the procedure itself generally yields limited therapeutic effects.

This patient presented to the hospital with syncope. The main etiologies of syncope in patients with HCM may include cardiac arrhythmias and primary hemodynamic abnormalities (8). HCM patients with syncope have an increased risk of sudden cardiac death (SCD). Guidelines from the European Society of Cardiology (ESC) and the American Heart Association (AHA) incorporate syncope into the decision-making considerations for implantable cardioverter defibrillator (ICD) implantation in patients with hypertrophic cardiomyopathy (5). Implantation of an ICD was recommended for this patient to prevent the risk of SCD, yet the patient declined the ICD implantation for the time being due to financial constraints.

In studies of patients with obstructive hypertrophic cardiomyopathy, mavacamten improves patients’ symptoms, exercise tolerance and quality of life, while also ameliorating LVOT obstruction, mitral regurgitation and diastolic function, and reversing adverse cardiac remodeling (9, 10). Consequently, mavacamten has now been approved for the treatment of obstructive HCM in multiple countries, with a Class I and Class IIa indication in relevant clinical guidelines.

In non-obstructive HCM, even in the absence of LVOT obstruction, patients still experience symptoms and impaired exercise capacity attributable to subendocardial ischemia, diastolic dysfunction, reduced left ventricular compliance and abnormal cellular energy metabolism. A small, phase 2, placebo-controlled trial in patients with non-obstructive HCM demonstrated that mavacamten can reduce levels of N-terminal pro-B-type natriuretic peptide (NT-proBNP) and cardiac troponin I (11). Additionally, one study showed that symptomatic patients with non-obstructive HCM treated with mavacamten exhibited improvements in left ventricular hypertrophy, left ventricular diastolic function and left atrial function at 48 weeks of treatment (12). None of these studies excluded patients with MVO.

A recently published case report described two patients with obstructive HCM complicated by MVO, who achieved a significant reduction in all pressure gradients with marked symptomatic improvement and normalization of NT-proBNP levels following mavacamten treatment (13). The mechanism underlying the therapeutic effect of mavacamten on MVO may be associated with the attenuation of excessive left ventricular myocardial contraction.

In summary, mavacamten is expected to provide a new therapeutic option for patients with HCM who are extremely difficult to treat. After the patient we reported received mavacamten treatment, echocardiography showed a significant reduction in the mid-left ventricular cavity pressure gradient, decreased levels of BNP and high-sensitivity cardiac troponin I, and marked relief of symptoms. No further syncope occurred during the treatment course, which confirms that mavacamten is effective in alleviating mid-left ventricular obstruction. Mavacamten therapy may be considered in patients with MVO to prevent the formation of apical ventricular aneurysms and the occurrence of SCD.

## Data Availability

The datasets presented in this study can be found in online repositories. The names of the repository/repositories and accession number(s) can be found at https://www.ncbi.nlm.nih.gov/snp/.
